# *Kalanchoe* sp. Extracts—Phytochemistry, Cytotoxic, and Antimicrobial Activities

**DOI:** 10.3390/plants12122268

**Published:** 2023-06-10

**Authors:** Justyna Stefanowicz-Hajduk, Anna Hering, Mariusz Kowalczyk, Rafał Hałasa, Magdalena Gucwa, J. Renata Ochocka

**Affiliations:** 1 Department of Biology and Pharmaceutical Botany, Medical University of Gdańsk, 80-416 Gdansk, Poland; anna.hering@gumed.edu.pl (A.H.); magdalena.gucwa@gumed.edu.pl (M.G.); renata@gumed.edu.pl (J.R.O.); 2 Department of Biochemistry and Crop Quality, Institute of Soil Science and Plant Cultivation, State Research Institute, 24-100 Pulawy, Poland; mkowalczyk@iung.pulawy.pl; 3 Department of Pharmaceutical Microbiology, Medical University of Gdańsk, 80-416 Gdansk, Poland; rafal.halasa@gumed.edu.pl

**Keywords:** cancer cell lines, Gram-positive bacteria, Gram-negative bacteria, *Candida albicans*, *Kalanchoe daigremontiana*, *Kalanchoe pinnata*, *Kalanchoe blossfeldiana*, water and dichloromethane fractions, antioxidant properties

## Abstract

*Kalanchoe* species are succulents occurring in tropical regions. They have many biological and pharmacological properties. In this study, the cytotoxic and antimicrobial activities of water and dichloromethane *Kalanchoe* fractions obtained from ethanol extracts of three *Kalanchoe* species—*K. daigremontiana*, *K. pinnata*, and *K. blossfeldiana* were estimated. The cytotoxic effect was assessed on human cancer cell lines—ovarian SKOV-3, cervical HeLa, breast MCF-7, and melanoma A375—by MTT (3-(4,5-dimethylthiazol-2-yl)-2,5-diphenyltetrazolium bromide) assay. The antimicrobial activity was estimated on selected Gram-positive and Gram-negative bacteria strains and on *Candida albicans*. The phytochemical analysis of selected *Kalanchoe* extracts was conducted by LC-QTOF-MS. The obtained results showed that the water fraction of *K. blossfeldiana* was active both on the tested cancer cells (IC_50_ values were 28.28 ± 2.76 and 32.51 ± 0.69 µg/mL on HeLa and SKOV-3, respectively) and bacteria strains (MIC values were 16 and 32 µg/mL on *S. epidermidis* and *S. aureus*, respectively). The water fraction of *K. pinnata* also had a significant effect on *S. epidermidis* and *S. aureus*, with MIC values of 32 and 64 µg/mL, respectively. The water fraction of *K. blossfeldiana* triggered a decrease in mitochondrial membrane potential (MMP) and induced cell cycle arrest in the G2/M phase in the SKOV-3 and HeLa cells. This fraction did not significantly increase cellular oxidative stress level. The DPPH and ABTS assays revealed that the water fraction of *K. blossfeldiana* had a strong antioxidant effect (IC_50_ was 9.44 ± 0.06 and 3.17 ± 0.1 µg/mL, respectively). The phytochemical analysis of the extracts of *K. blossfeldiana* and *K. pinnata* revealed the presence of at least 218 main components. The most frequently occurring were flavonol glycosides (31 metabolites), phenylpropanoids (13 metabolites), gallic acid derivatives (13 compounds), benzoic acid derived compounds (14 metabolites), and acyclic alcohol glycosides (16 compounds). In addition, proanthocyanidins were detected mainly in *K. blossfeldiana.* The study indicates that the water fraction of *K. blossfeldiana* has significant biological potential and can be further investigated towards anticancer and antimicrobial application.

## 1. Introduction

*Kalanchoe* species (syn. *Bryophyllum*) from the Crassulaceae family are perennial plants found in tropical areas. This genus consists of approximately 150 species, most of them growing in Africa, Brazil, and India. The well-known species are *K. pinnata*, *K. daigremontiana*, *K. gracilis*, *K. brasiliensis*, *K. blossfeldiana*, *K. hybrida*, *K. crenata*, and *K. tubiflora*. Extracts of most of these plants are used to treat allergies, internal and skin infections, asthma, otitis, and headaches [1]. Ethnomedicinal uses in different countries indicate that *Kalanchoe* species are applied in chickenpox, stomach aches, and fevers in the USA [2]. In Brazil, the plants are used in treating abscesses, arthritis, bronchitis, coughs, dermatitis, eczema, glaucoma, insect stings, and rheumatism [2]. *Kalanchoe* plants are also well-known in the treatment of abdominal discomfort, diabetes, dysentery, wounds, eye infections, menstrual disorders, ear aches, nausea, and urethritis in India, Mexico, and Peru [2,3,4]. It should be emphasized that *Kalanchoe* plants are toxic to cattle and other farm stocks, contributing to heart rate abnormalities, diarrhea, anorexia, and even death [5,6]. These symptoms are caused by the presence of the main plant metabolites—bufadienolides, the amount of which is different depending on the species. This group of phytochemicals has been used in cardiac dysfunction due to their strong effect on the heart. They increase intracellular calcium ion concentration, act as inhibitors of the Na^+^/K^+^-ATPase pump, and increase the contractile force of the heart [7,8,9,10]. However, the *Kalanchoe* plants have many other biological and pharmacological activities [11]. Among them, the most important are immunomodulatory, antibacterial, antiviral, antiproliferative, antihistaminic, and antidiabetic effects [5,12,13,14,15,16,17].

Generally, the medicinal properties of these plants result not only from the content of bufadienolides but also from the presence of flavonoids. Among the flavonoid compounds are kaempferol, quercetin, patuletin, luteolin, eupafolin, quercitrin, and isorhamnetin derivatives [5,18,19,20,21,22]. Among the bufadienolides, bersaldegenin, bryophyllin, helebrigenin, kalanhybrin, daigremontianin, daigredorigenin, kalanchosides, and their derivatives are present. Other metabolites were also detected in *Kalanchoe* species, such as anthocyanins, coumarins, phenolic acids, sterols, and fatty acids [5,10,23,24,25,26,27,28].

An interest in the biological and pharmacological properties of *Kalanchoe* species is continually growing, but there are still many unknowns regarding the effects of extracts in the treatment of cancer and bacterial infections. In this study, the cytotoxic and antimicrobial activities of water and dichloromethane fractions of *K. daigremontiana*, *K. pinnata*, and *K. blossfeldiana* were estimated.

## 2. Results

### 2.1. Anticancer Study

#### 2.1.1. Cytotoxic Activity

To estimate the cytotoxic effect of the obtained water and dichloromethane fractions, we performed MTT test on four cancer cell lines—human ovarian SKOV-3, cervical HeLa, breast MCF-7, and melanoma A375 (Table 1). The obtained results showed that the most active fraction was the dichloromethane fraction of *K. daigremontiana*, with IC_50_ range values of 5.42–8.02 µg/mL for all the cells. Significant activity was also observed for the water fraction of *K. blossfeldiana*, with IC_50_ values of 28.28 ± 2.76 and 32.51 ± 0.69 µg/mL for HeLa and SKOV-3 cells, respectively, and for the dichloromethane fraction of *K. pinnata*, with an IC_50_ value of 32.10 ± 0.73 µg/mL for SKOV-3 cells. The other IC_50_ values of the tested fractions were equal to or greater than 50 µg/mL.

The cytotoxic activity of dichloromethane fraction of *K. daigremontiana* has been already described in the previous work [22]. Since the water fraction of *K. blossfeldiana* also turned out to be interesting in terms of the cytotoxic effect, we selected this fraction for further anticancer study.

**Figure 1:**
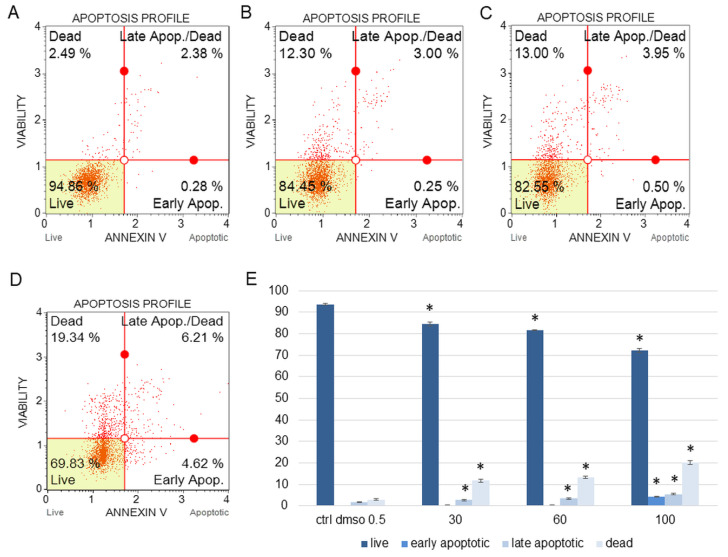
The percentages of live, apoptotic, and dead SKOV-3 cells after treatment with the water fraction of *Kalanchoe blossfeldiana*. The cells were treated with DMSO (0.5%, *v*/*v*, (**A**)) and the fraction at concentrations of 30 (**B**), 60 (**C**), and 100 µg/mL (**D**) for 24 h. The analysis was performed by flow cytometry. All the results are shown in the graph (**E**) and compared with the control. Each sample was run at least in two independent repeats (in three repetitions). Error bars indicate standard deviations. Significant differences relative to the control are marked with an asterisk “*” (*p* < 0.05).

**Figure 2:**
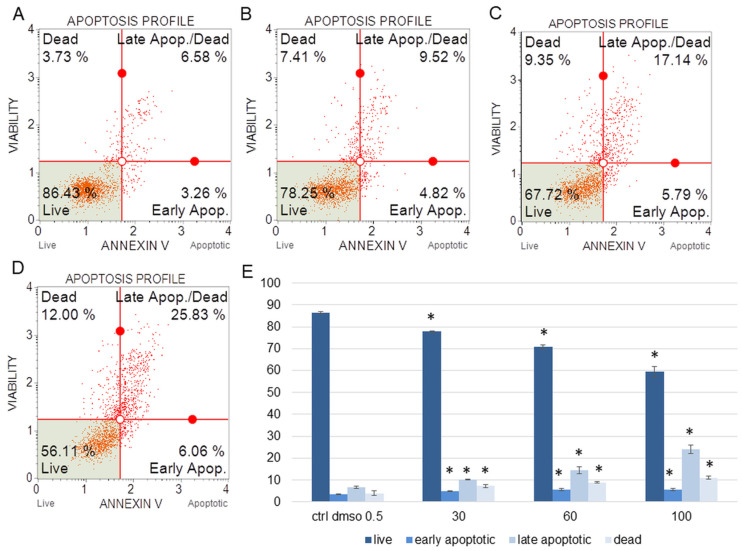
The percentages of live, apoptotic, and dead HeLa cells after treatment with the water fraction of *Kalanchoe blossfeldiana*. The cells were treated with DMSO (0.5%, *v*/*v*, (**A**)) and the fraction at concentrations of 30 (**B**), 60 (**C**), and 100 µg/mL (**D**) for 24 h. The analysis was performed by flow cytometry. All the results are shown in the graph (**E**) and compared with the control. Each sample was run at least in two independent repeats (in three repetitions). Error bars indicate standard deviations. Significant differences relative to the control are marked with an asterisk “*” (*p* < 0.05).

The water fraction of *K. blossfeldiana* was evaluated on the SKOV-3 (Figure 1) and HeLa (Figure 2) cells to estimate the percentage of apoptotic and dead cells after treatment of these cells with the fraction for 24 h. The results in the experiment with annexin and 7-AAD showed that the water fraction of *K. blossfeldiana* was cytotoxic in a dose-dependent manner on both cell lines.

The amount of early apoptotic SKOV-3 and HeLa cells did not significantly increase in comparison with the control. The percentages of late apoptotic SKOV-3 cells were 1.82 ± 0.31, 2.69 ± 0.28, 3.43 ± 0.40, and 5.46 ± 0.75% for the control (DMSO), 30, 60, and 100 µg/mL of the fraction, respectively (Figure 1). In the case of the HeLa cells, the amounts of late apoptotic cells significantly increased and were 6.48 ± 0.14, 10.24 ± 1.56, 15.28 ± 2.03, 23.93 ± 1.64% for the control (DMSO), 30, 60, and 100 µg/mL of the fraction, respectively (Figure 2). The amounts of dead SKOV-3 cells were 2.97 ± 0.69, 11.70 ± 0.6, 13.34 ± 0.76, 20.13 ± 0.25%, while in the experiments with the HeLa cells, the results were 3.92 ± 0.81, 7.21 ± 0.32, 8.85 ± 0.65, 10.98 ± 1.37% for the control (DMSO), 30, 60, and 100 µg/mL of the fraction, respectively.

#### 2.1.2. The Effect of the Water Fraction of *K. blossfeldiana* on Mitochondrial Membrane Potential (MMP), Oxidative Stress Level, and Cell Cycle Arrest in SKOV-3 and Hela Cells

**Figure 3:**
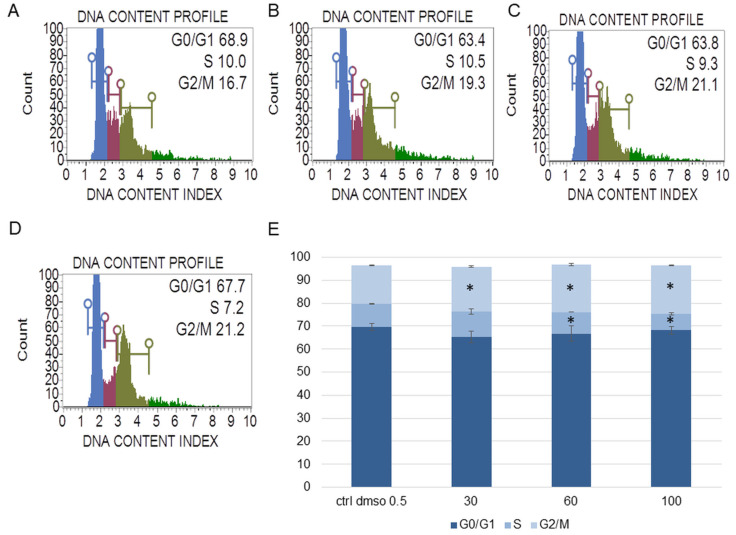
The effect of the water fraction of *Kalanchoe blossfeldiana* on cell cycle in SKOV-3 cells after 48 h of treatment. The cells were treated with DMSO (0.5%, *v*/*v*, (**A**)) and the fraction at concentrations of 30 (**B**), 60 (**C**), and 100 µg/mL (**D**) and analyzed by flow cytometry. Blue color—G0/G1, purple—S, and green—G2/M phase. The percentage of the cells in each phase was determined in comparison with the control (**E**). The experiment was repeated in two independent repeats (in three repetitions). Error bars represent standard deviations. Significant differences relative to the control are marked with an asterisk “*” (*p* < 0.05).

**Figure 4:**
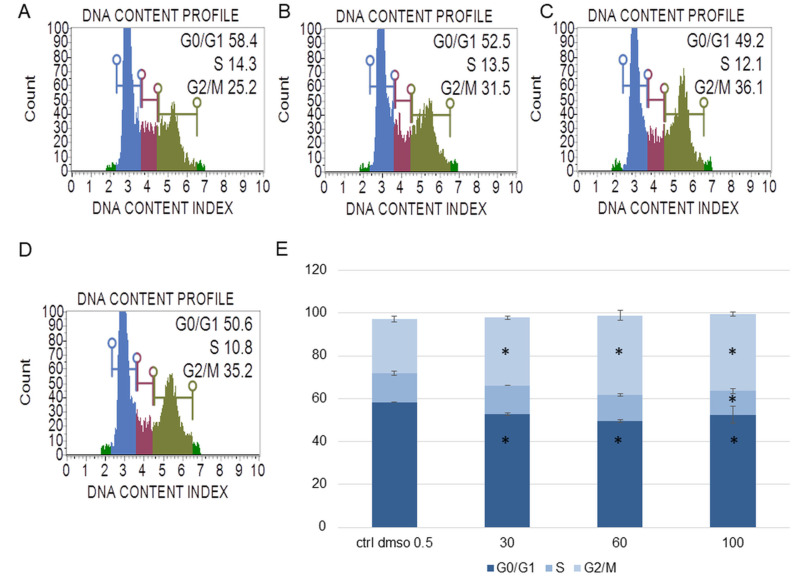
The effect of the water fraction of *Kalanchoe blossfeldiana* on cell cycle in HeLa cells after 48 h of treatment. The cells were treated with DMSO (0.5%, *v*/*v*, (**A**)) and the fraction at concentrations of 30 (**B**), 60 (**C**), and 100 µg/mL (**D**) and analyzed by flow cytometry. Blue color—G0/G1, purple—S, and green—G2/M phase. The percentage of the cells in each phase was determined in comparison with the control (**E**). The experiment was repeated in two independent repeats (in three repetitions). Error bars represent standard deviations. Significant differences relative to the control are marked with an asterisk “*” (*p* < 0.05).

**Figure 5:**
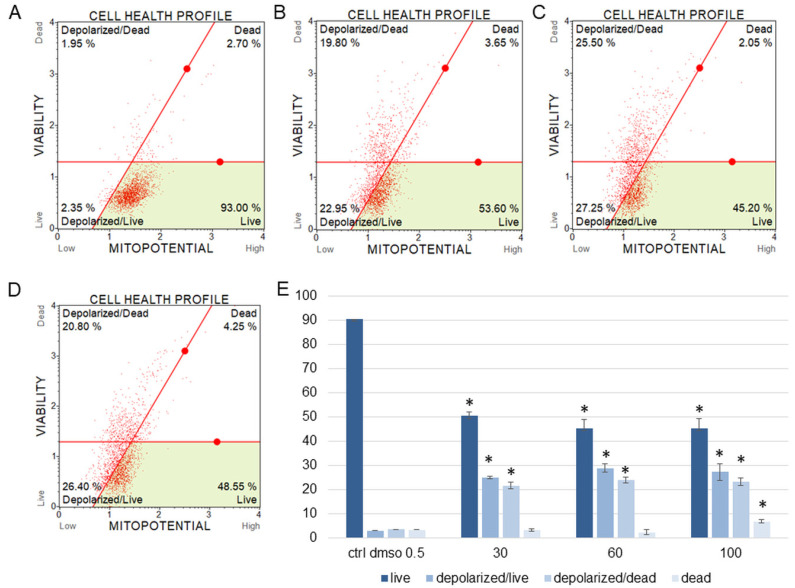
Mitochondrial membrane potential changes in SKOV-3 cells treated with the water fraction of *Kalanchoe blossfeldiana*. The cells were treated with DMSO (0.5%, *v*/*v*, (**A**)) and the fraction at concentrations of 30 (**B**), 60 (**C**), and 100 µg/mL (**D**) for 24 h. The analysis was performed by flow cytometry. The percentages of live, depolarized/live, depolarized/dead, and dead cells are shown in the graph (**E**) and compared with the control. Each sample was run at least in two independent repeats (in three repetitions). Error bars indicate standard deviations. Significant differences relative to the control are marked with an asterisk “*” (*p* < 0.05).

**Figure 6:**
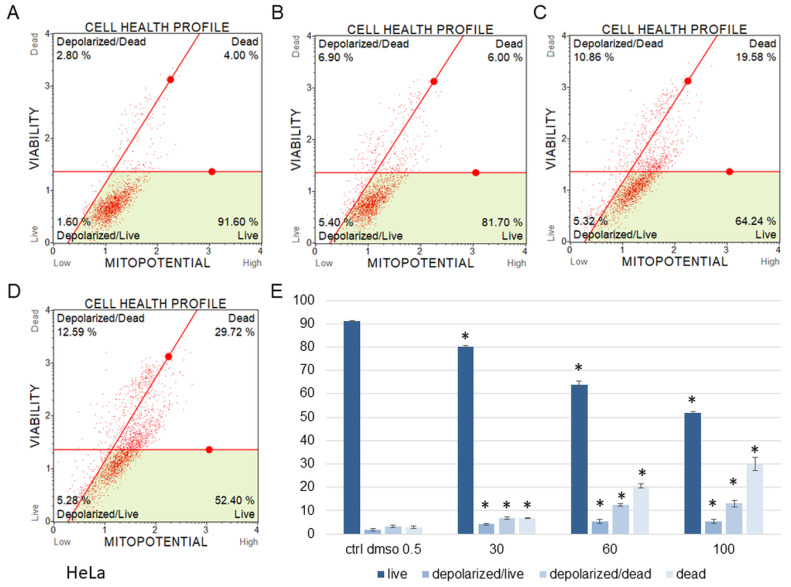
Mitochondrial membrane potential changes in HeLa cells treated with the water fraction of *Kalanchoe blossfeldiana*. The cells were treated with DMSO (0.5%, *v*/*v*, (**A**)) and the fraction at concentrations of 30 (**B**), 60 (**C**), and 100 µg/mL (**D**) for 24 h. The analysis was performed by flow cytometry. The percentages of live, depolarized/live, depolarized/dead, and dead cells are shown in the graph (**E**) and compared with the control. Each sample was run at least in two independent repeats (in three repetitions). Error bars indicate standard deviations. Significant differences relative to the control are marked with an asterisk “*” (*p* < 0.05).

**Figure 7:**
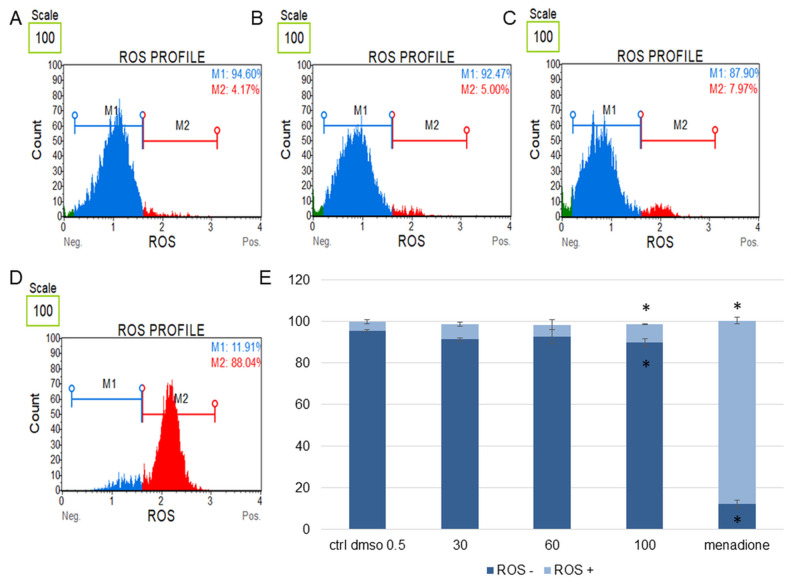
Changes in the level of oxidative stress in SKOV-3 cells treated with the water fraction of *Kalanchoe blossfeldiana*. The cells were treated with DMSO (0.5%, *v*/*v*, (**A**)) and the fraction at concentrations of 60 (**B**), 100 µg/mL (**C**), and menadione at 200 µM (D) for 24 h. The analysis was performed by flow cytometry. The percentages of ROS negative (ROS−) and ROS positive (ROS+) cells are shown in the graph (**E**) and compared with the control. Menadione was a positive control. Each sample was run at least in two independent repeats (in three repetitions). Error bars indicate standard deviations. Significant differences relative to the control (DMSO) are marked with an asterisk “*” (*p* < 0.05).

**Figure 8:**
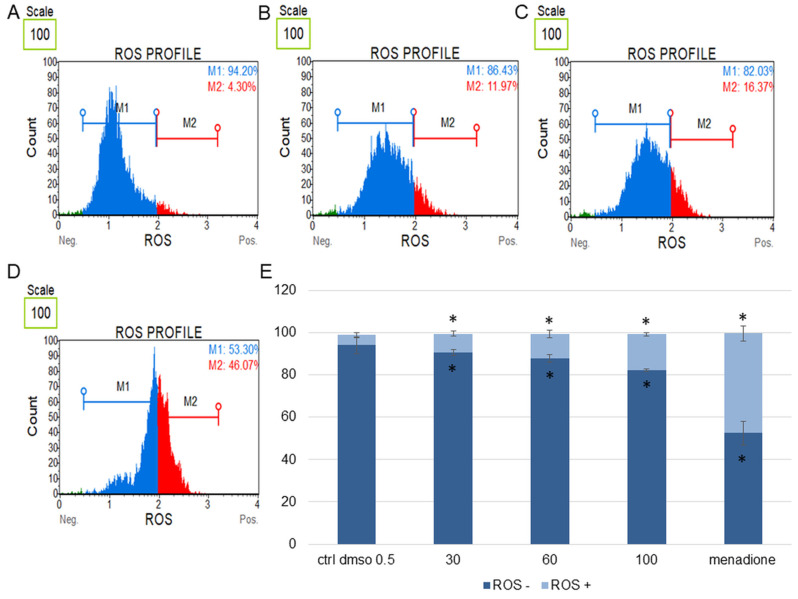
Changes in the level of oxidative stress in HeLa cells treated with the water fraction of *Kalanchoe blossfeldiana*. The cells were treated with DMSO (0.5%, *v*/*v*, (**A**)) and the fraction at concentrations of 60 (**B**), 100 µg/mL (**C**), and menadione at 200 µM (**D**) for 24 h. The analysis was performed by flow cytometry. The percentages of ROS negative (ROS−) and ROS positive (ROS+) cells are shown in the graph (**E**) and compared with the control. Menadione was a positive control. Each sample was run at least in two independent repeats (in three repetitions). Error bars indicate standard deviations. Significant differences relative to the control (DMSO) are marked with an asterisk “*” (*p* < 0.05).

In the next step, we determined the effect of the water fraction of *K. blossfeldiana* on cell cycle arrest, mitochondrial potential in the cells, and changes in the level of cellular oxidative stress (Figure 3, Figure 4, Figure 5, Figure 6, Figure 7 and Figure 8).

The experiments showed that the water fraction of *K. blossfeldiana* triggered cell cycle arrest in G2/M in the SKOV-3 (Figure 3) and HeLa cells (Figure 4). In the SKOV-3 cells, the percentage of the cells in the control (DMSO) in G2/M phase was 16.80 ± 0.14 and then 19.73 ± 0.38, 20.60 ± 0.56, 21.17 ± 0.15 for the water fraction of *K. blossfeldiana* at concentrations of 30, 60, and 100 µg/mL, respectively. For the HeLa cells, the changes were more significant, and the percentage of the cells in G2/M was 25.33 ± 1.31 for the control (DMSO) and then 31.67 ± 0.86, 37.13 ± 2.42, and 35.90 ± 0.99 for the water fraction of *K. blossfeldiana* at concentrations of 30, 60, and 100 µg/mL, respectively.

The results obtained from MMP assay indicated that the amount of depolarized/live SKOV-3 cells was significantly increased after 24 h of the cell incubation with the fraction in comparison with the control (Figure 5). These amounts were 2.93 ± 0.46, 24.95 ± 1.78, 28.75 ± 3.47, 27.15 ± 2.47% for the control (DMSO), 30, 60, and 100 µg/mL of the fraction, respectively. The percentages of depolarized/dead cells were similar and were 3.45 ± 1.48, 21.60 ± 1.33, 23.88 ± 1.52, 23.05 ± 1.57% for the control (DMSO), 30, 60, and 100 µg/mL of the fraction, respectively. In the experiments with the HeLa cells, the number of depolarized/live cells did not increase as significantly as in the SKOV-3 cells, and the results were 1.79 ± 0.33, 4.16 ± 1.04, 5.35 ± 0.76, 5.38 ± 0.25% for the control (DMSO), 30, 60, and 100 µg/mL of the fraction, respectively (Figure 6). However, the percentages of depolarized/dead cells were much increased in the HeLa cells in comparison with the control and were 3.24 ± 0.53, 6.73 ± 0.47, 12.35 ± 1.41, 13.10 ± 0.51% for the control (DMSO), 30, 60, and 100 µg/mL of the fraction, respectively. In both cell lines, we observed different effects of the water fraction of *K. blossfeldiana* on the mitochondrial potential.

In this study, the levels of cellular oxidative stress in the SKOV-3 (Figure 7) and HeLa cells (Figure 8) treated with the *K. blossfeldiana* water fraction for 24 h were estimated.

The obtained data indicated that this fraction did not significantly change the level of oxidative stress in the tested cell lines in comparison with the negative (0.5% DMSO) and positive controls (menadione at concentration of 200 µM). The increase in ROS (reactive oxygen species) level was more pronounced in the HeLa cells (Figure 8) than in SKOV-3 (Figure 7). The results for the HeLa ROS+ cells were 4.81 ± 0.83, 9.02 ± 1.89, 11.60 ± 0.73, 17.02 ± 3.59% for the control (DMSO), 30, 60, and 100 µg/mL of the fraction, respectively. In the case of the SKOV-3 cells the percentages of ROS+ cells were 4.16 ± 0.85, 7.37 ± 2.41, 5.64 ± 0.09, 8.93 ± 1.72% for the control (DMSO), 30, 60, and 100 µg/mL of the fraction, respectively.

The anticancer study revealed that the water fraction of *K. blossfeldiana* did not have the ability to generate much oxidative stress in cancer cells. Thus, additional experiments—DPPH and FRAP assays—were performed, in which the antioxidant potential of this water fraction was estimated (Table 2).

The water fraction of *K. blossfeldiana* exhibited strong antioxidant activity, and this effect was higher in comparison with ascorbic acid in both tests.

### 2.2. Microbiological Study

In this work, we performed microbiological experiments with the obtained water and dichloromethane fractions of *Kalanchoe* species and ten bacteria strains—*Streptococcus* β-hemolyzing group A PCM465, *Streptococcus* β-hemolyzing group G, *Corynebacterium diphtheriae*, *Staphylococcus aureus* ATCC6538, *Staphylococcus epidermidis* ATCC14990, *Helicobacter pylori* ATCC43504, *Cutibacterium acnes* ATCC6919 (*Propionibacterium acnes*), *Streptococcus equinus* ATCC15351, *Clostridium bifermentans* ATCC638, and *Clostridium sporogenes* ATCC19404—and of *Candida albicans* ATCC10231 (Table 3). The MIC values for all the tested fractions were in the range of 16 µg/mL–15 mg/mL. The most active fractions were the water fractions of *K. blossfeldiana*, *K. pinnata*, and *K. daigremontiana* and the dichloromethane fraction of *K. blossfeldiana*. These fractions were effective on *Staphylococcus aureus*, with MIC values of 32 and 64 µg/mL for the water fractions of *K. blossfeldiana* and *K. pinnata*, respectively. In the experiments with *Staphylococcus epidermidis*, the MIC values were 16, 32, 64, and 64 µg/mL for the water fractions of *K. blossfeldiana*, *K. pinnata*, *K. daigremontiana* and the dichloromethane fraction of *K. blossfeldiana*, respectively. The other obtained MIC and MBC values of the fractions did not indicate a significant effect of the fractions on bacteria strains or *Candida albicans*.

### 2.3. Phytochemical Profiles of the Water Fractions of K. blossfeldiana and K. pinnata Obtained by LC-QTOF-MS

For the phytochemical analysis, we selected the water fraction of *K. blossfeldiana* and *K. pinnata* from all obtained extracts, which were active both on cancer cells and bacteria strains.

High-resolution mass spectrometry data analysis revealed the presence of at least 218 main components in the investigated samples. On the basis of the MS2 spectra acquired at 3 collision energy levels, 218 compounds were detected, and from them, 150 were classified into 26 arbitrarily established general compound categories (Table 4, Figures S1, S2 and Table S1 in Supplementary Materials).

The most frequently occurring in the investigated samples were flavonol glycosides (31 metabolites). However, most flavonol glycosides were detected only in the *K. pinnata* water phase fraction and sporadically observed in the water fraction of *K. blossfeldiana*. Most of the detected flavonol glycosides were derived from quercetin, kaempferol, or gossypetin, with relatively simple and short glycoside components consisting of pentoses, deoxyhexoses, hexoses, and, less frequently, hexuronic acids.

The acyclic alcohol glycosides (16 compounds) comprised the group most challenging to identify using the mass spectrometric methods due to their very similar fragmentation patterns. Most class members were detected in the *K. pinnata* water fraction, with octenyl pentose–hexoside tentatively identified as this group’s main component.

Benzoic acid derived compounds were also abundantly present in the *Kalanchoe* extracts (14 metabolites) but, again, only sporadically detected in the sample originating from *K. blossfeldiana*. As in the case of the structurally related gallic acid derivatives, they were observed mainly as simple glycosides, presumably glucosides (although the exact identity of the attached carbohydrate was not established experimentally).

The next largest group was phenylpropanoids, observed mainly as glycosides, coumaric, caffeic, and ferulic acids (13 metabolites). Similar to the previous group of compounds, a greater variety of detected phenylpropanoid derivatives was observed in the *K. pinnata* water fraction. On the basis of the results of semi-quantitation, the highest contents were observed for various hexosides of coumaric acid, presumably with both ester- and ether- linkages.

The investigated samples were also rich in gallic acid derivatives (13 compounds). Among these were mainly simple glycosides, such as 1-O-glucoside or glucogallin, observed in two peaks, presumably due to acyl migration. We also detected rather unusual sulfo-hexose and quinic acid derivatives of gallic acid. Unlike in the case of the previous two groups of compounds, a greater variety and higher estimated contents of gallic acid related compounds were observed in the *K. blossfeldiana* fraction.

Also worth mentioning are acyclic nitrile glycosides, known to occur in at least some Crassulaceae members and detected in samples from both the investigated species. The group of acyclic nitrile glycosides can be further divided into cyanogenic and non-cyanogenic sub-groups. The former consists of α-nitrile glycosides (e.g., compounds where both nitrile and hydroxyl group-forming O-glycosidic bonds are attached to the same carbon atom), represented in the studied samples by lotaustralin and sachaloside V. The non-cyanogenic group is represented by β-nitrile rhodiocyanoside D (or its isomeric γ-nitrile glycoside rhodiocyanoside A) and γ-nitrile, sarmentosin. On the basis of semi-quantitative data, the acyclic nitrile glycosides were found to be among the main components of the studied extracts.

Proanthocyanidins were more prominently observed in the sample originating from *K. blossfeldiana* and were almost absent in *K. pinnata*. Although mostly dimeric proanthocyanidins were detected, on the basis of the signal from the CAD detector, we speculated that congeners with a higher degree of polymerization were also present in the *K. blossfeldiana* sample but were not detected by mass spectrometry due to poor ionizability.

Samples from both species also contained many megastigmane (sesquiterpene) glycosides, similar to acyclic alcohol glycosides, which are inherently difficult to identify without the appropriate reference standards.

The members of the Crassulaceae family are known to accumulate large amounts of sedoheptulose, which was also observed in the studied samples. Another distinct feature of the metabolism of Crassulaceae plants is an accumulation of iso-citrate, although we could not entirely distinguish it from citrate on the basis of the MS data alone.

**Figure 9:**
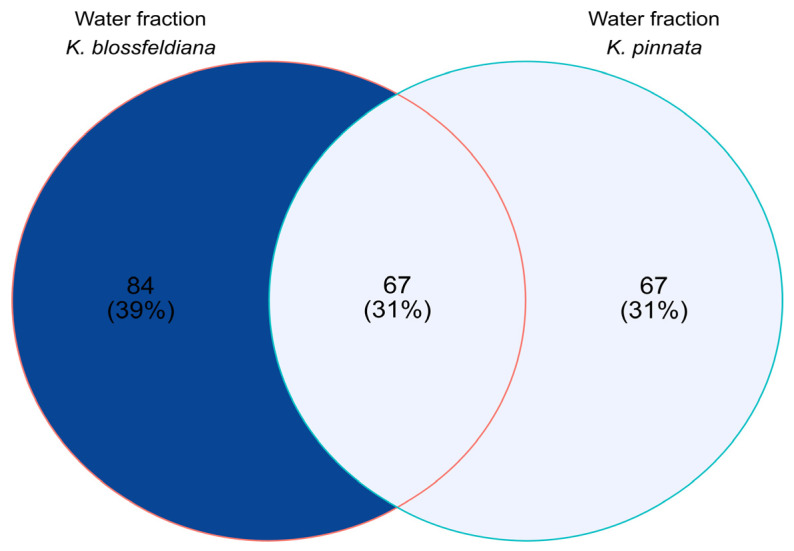
Venn diagram based on qualitative analysis of the water fractions of *Kalanchoe blossfeldiana* and *Kalanchoe pinnata*.

The commonalities and differences in the number of detected compounds between analyzed samples are shown in Figure 9.

## 3. Discussion

In this study, the anticancer and antimicrobial effects of water and dichloromethane fractions obtained from *K. daigremontiana*, *K. pinnata*, and *K. blossfeldiana* were estimated. The cytotoxic study revealed that the most active from the tested fractions were the dichloromethane fractions of *K. daigremontiana* and *K. pinnata* and the water fractions of *K. blossfeldiana* and *K. pinnata*. The last two fractions were also active on *S. epidermidis* and *S. aureus*. For further anticancer study, we chose the water fraction of *K. blossfeldiana* due to the fact that this fraction exerted significant activity on the ovarian and cervical cancer cells, and so far its effect on MMP changes, generation of oxidative stress, and cell cycle has not been described. In the previous study on cytotoxic activity of water and ethanol extracts of *Kalanchoe* species, the best in vitro anticancer effect we observed was for the ethanol extract of *K. blossfeldiana* and the water extract of *K. daigremontiana* [22,29]. In the present study with the SKOV-3 and HeLa cells, the water fraction of *K. blossfeldiana* triggered cell death and cell cycle arrest in G2/M; however, in the HeLa cells, this effect was more pronounced. In the HeLa cells, the quantity of apoptotic cells was much higher at the tested concentrations of the fraction than in the SKOV-3 cells. These results were consistent with data obtained in the assay that estimated MMP changes in both cell lines. In the case of SKOV-3, we observed a significant decrease in MMP in live cells, while in the HeLa cells, the populations of depolarized/dead cells were mostly detected.

Mitochondria are a great target for studying the toxic effect of many xenobiotics due to the fact that they play a vital role in cellular physiology. They generate the majority of the cellular energy (ATP) through oxidative phosphorylation [30]. A series of redox reactions in mitochondria creates an electrochemical gradient and generates the mitochondrial membrane potential (MMP) [31]. Mitochondrial dysfunctions are associated with the influence of many xenobiotics on cells and their role in various disorders, such as neurodegenerative diseases, diabetes, cancer, and cardiovascular diseases [32]. These compounds may change MMP by perturbing a variety of macromolecules in the mitochondria. A decrease in the MMP may also be linked to apoptosis [33].

Oxidative stress is also associated with cell death. Generally, reactive oxygen species (ROS) are produced mainly by mitochondria as products of oxygen metabolism and can play several physiological roles (i.e., cell signaling) [34]. An increase in ROS production is observed in cells treated with UV, heavy metals, ionizing radiation, and xenobiotics. These changes in the ROS level can negatively affect several cellular structures—e.g., proteins, lipids, and DNA—and lead to cell death [34,35,36,37,38,39]. In this work, we estimated the role of the water fraction of *K. blossfeldiana* on reactive oxygen species generation in the SKOV-3 and HeLa cells. The obtained results showed that the tested fraction slightly increased the level of cellular ROS in both the SKOV-3 and HeLa cells, but in the HeLa cells, this effect was more pronounced. Thus, in the next step, we also performed the antioxidant assays with the water fraction of *K. blossfeldiana*, in which we showed that this fraction had a strong antioxidant effect and potentially may be used in cancer protection. However, further study should be undertaken on this topic.

The cytotoxic activity was evaluated on different *Kalanchoe* species. The methanol extract of *K. hybrida* showed significant activity on breast cancer MCF-7 cells and lung carcinoma NCI-H460 cells [28]. In addition, water and alcohol extracts of *K. thrysiflora* and *K. marmorata* and their fractions (n-butanol, methylene chloride, and ethyl acetate) were examined on the MCF-7 cells. The methylene chloride fraction was the most active on the cells [14]. Bufadienolides present in *Kalanchoe* species can be partly responsible for the cytotoxic activity of the plant extracts. For example, kalanchosides from the aerial parts of *K. gracilis* showed an antiproliferative effect on a panel of human tumor cell lines, with potency reaching the nanomolar range [27,40]. Han et al. tested a few bufadienolides (arenobufagin, bufotalin, hellebrigenin, hellebrigenol, and bufalin) on human liver cancer cells (HepG2). These compounds had significant antiproliferative effects at concentrations below 150 ng/mL [41]. Furthermore, arenobufagin and hellebrigenin were tested on the human glioblastoma cell line U-87 [42]. The inhibitory effect of bufalin and cinobufagin was shown on the proliferation of androgen-dependent (LNCaP) and -independent prostate cancer cell lines (DU145 and PC3) [43]. The previous study on bersaldegenin-1,3,5-orthoacetate revealed its strong anticancer effect in vitro, with an IC_50_ value of approximately 1.0 µg/mL towards the SKOV-3, HeLa, MCF-7, and A375 cells [22,44].

In addition to anticancer results, we obtained promising data in the microbiological experiments. The most active fractions were the water fraction of *K. blossfeldiana* and *K. pinnata* on *S. aureus* and *S. epidermidis*. Less active, but still strong were the water fraction of *K. daigremontiana* and dichloromethane fraction of *K. blossfeldiana* on *S. epidermidis*. These results confirm the ethnomedicinal uses of *Kalanchoe* species in bacterial infections, especially in the cases related to the skin.

Methanol extract from leaves of *K. pinnata* was investigated on *Bacillus subtilis*, *S. aureus*, *Shigella dysenteriae*, *Proteus vulgaris*, and *Escherichia coli*, and it inhibited the growth of these strains [45]. Other extracts of *K. pinnata* and *K. crenata* (water, methanol, palm wine, gin, and raw juice) were also tested on Gram-positive and Gram-negative bacteria strains [46]. In addition, *K. daigremontiana* water, n-hexane, carbon tetrachloride, and chloroform fractions were tested, among others, on *Bacillus subtilis*, *S. aureus*, *Escherichia coli*, *Pseudomonas aeruginosa*, *Shigella dysenteriae*, *Vibrio mimicus*, *Candida albicans*, and *Aspergillus niger*. The best activity was shown for the carbon tetrachloride fraction [15]. In the previous study with water and ethanol extracts from *K. daigremontiana*, *K. pinnata*, and *K. blossfeldiana*, the best activites on bacteria strains were for ethanol extracts of *K. blossfeldiana* and *K. pinnata* on the methicillin-resistant *S. aureus* (MRSA) clinical strain, *S. epidermidis* and *Enterococcus hirae* [29].

The cytotoxic and antibacterial activities of the water fraction of *K. blossfeldiana* result from the variety of its phytochemical composition. In this work, we compared the contents of secondary metabolites of *K. blossfeldiana* with *K. pinnata*. The phytochemical analysis revealed that the most frequently detected groups of compounds in the *K. pinnata* water phase fraction were flavonol glycosides and phenylpropanoids, observed mainly as glycosides, coumaric, caffeic, and ferulic acids. In addition, benzoic acid derived compounds were abundantly present in *K. pinnata* fraction. In contrast, a greater variety and higher estimated contents of gallic acid related compounds were observed in *K. blossfeldiana* than in *K. pinnata*. Also, proanthocyanidins were more prominently observed in the sample originating from *K. blossfeldiana*, being almost absent in *K. pinnata*. The study on *Kalanchoe* fractions revealed that, in the case of cytotoxic and antimicrobial activities of the water fraction of *K. blossfeldiana*, compounds related to gallic acid and proanthocyanidins may have played a key role, while in the biological action of the water fraction of *K. pinnata*, flavonol glycosides, phenylpropanoids, acyclic alcohol glycosides, and benzoic acid derived compounds were of great importance. Moreover, the extracts from both plant species contained large amounts of sarmentosin, which was approximately twofold higher in the *K. pinnata* fraction than in *K. blossfeldiana*. This compound has not been described in these plants nor have organic acids (citrate, aconitate, isocitrate, homocitrate, malate, or ascorbate). Among the phenylpropanoid and benzoic acid derived compounds—melilotoside, the coumaroyl, saliciloyl, and hydroxymethoxybenzoyl derivatives, respectively, have also not been demonstrated in *K. pinnata* and *K. blossfeldiana*. The same situation occurred with megastigmane (megastigmanen, megastigmenon, and iondiolone), acyclic alcohol and acyclic nitrile (lotaustralin and rhodiocyanoside) derivatives. In turn, flavonol glycosides are well-known compounds in *Kalanchoe* species, especially in *K. pinnata* (quercetin and kaempferol derivatives) [47,48]; however, the presence of gossypetin glycosides has not been described in this plant. Kaempferol and quercetin with pelargonidin, cyanidin, peonidin, delphinidin, petunidin, and malvidin-derived compounds were analyzed in *K. blossfeldiana* flowers [49]. Epigallocatechin gallate and gallic acid have already been detected in the leaves of *K. blossfeldiana* [50,51]. In turn, referring to the chemical composition of *K. daigremontiana* water fraction described in the previously published work [22], this extract contained mostly flavonol glycosides—quercetin, kaempherol, patuletin, myricetin and isorhamnetin derivatives—and isoflavones. In this case, flavonoid compounds may be responsible for the activity of this water fraction on *S. epidermidis*; however, no significant effect was observed on the cancer cells. On the other hand, the most cytotoxically active dichloromethane fraction of *K. daigremontiana* contained bufadienolides [22].

## 4. Materials and Methods

### 4.1. Preparation of Plant Fractions

Fresh leaves of *K. daigremontiana*, *K. pinnata*, and *K. blossfeldiana* were purchased from a commercial garden source (Garden Center Justyna, Gdansk, Poland). Voucher specimens (No. 21761-21763 for *K.d*., *K.p.*, and *K.b*., respectively) were deposited in the Herbarium of the Medical University of Gdańsk (GDMA Herbarium). The water and dichloromethanol fractions were prepared from ethanol extracts. The leaves of three species (100 g) were macerated and stirred with 95% ethanol (0.5 L) for 24 h at RT. The ethanol extracts were filtered, concentrated under reduced pressure at 40 °C, and shaken 3 times with water and dichloromethane. The obtained fractions (from the same ethanol extracts) were divided, concentrated under reduced pressure at 40 °C, and lyophilized. The fractions were dissolved in a sterile dimethyl sulfoxide (DMSO, 20 mg/mL) for cytotoxic and microbiological tests.

### 4.2. Anticancer Study

#### 4.2.1. Cell Culture

The human ovarian cancer (SKOV-3), malignant melanoma (A375), cervical adenocarcinoma (HeLa S3), and breast cancer (MCF-7) cell lines were obtained from the American Type Culture Collection (ATCC, Manassas, VA, USA). The A375, HeLa S3, and MCF-7 cell lines were cultured in Dulbecco’s Modified Eagle’s Medium (DMEM, Merck Millipore, Burlington, MA, USA). The SKOV-3 line was cultured in McCoy’s Medium. Both media were supplemented with 100 units/mL of penicillin, 100 mg/mL of streptomycin, and 10% (*v*/*v*) foetal bovine serum (FBS). The cells were incubated at 37 °C and 5% CO_2_.

#### 4.2.2. Cytotoxic Assays

##### MTT Test

To estimate the inhibition of cell proliferation and viability, we performed MTT assay [52]. The cell lines were seeded in 96-well plates (5 × 10^3^ cells/well) and treated with the water or dichloromethane plant fractions at a concentration range of 2.0–150 µg/mL. The DMSO concentration used in the control sample was 0.75% (*v*/*v*). After 24 h, MTT (0.5 mg/mL) was added to the cells for 3 h. The absorbance of the formazan solution (in DMSO) was measured on a microtiter plate reader (Epoch, BioTek Instruments, Winooski, VT, USA). All data were analysed in GraFit software v.7 (Erithacus Software, East Grinstead, West Sussex, UK) and were expressed as IC_50_ mean values (± standard deviation, SD) of three independent experiments (in six repetitions, *n* = 18).

##### Annexin V and Dead Cell Assay

The SKOV-3 and HeLa cells (1 × 10^5^ cells/well) were seeded in 12-well plates and incubated for 24 h. After this time, the water fraction of *K. blossfeldiana* was added to the cells at concentrations of 30, 60, and 100 µg/mL. The control was the cell group with 0.5% DMSO (*v*/*v*). After 24 h, the cells were washed with PBS and stained with fluorescent reagents (annexin V and 7-AAD (7-aminoactinomycin)), according to the manufacturer’s instruction [53]. The stained cells were analyzed by flow cytometry (Muse Cell Analyzer, Merck Millipore, Burlington, MA, USA). The experiments were performed in at least two independent repeats (in three repetitions).

##### Mitochondrial Potential Assay

The SKOV-3 and HeLa cells (1 × 10^5^ cells/well) were seeded in 12-well plates and incubated for 24 h. After this time, the water fraction of *K. blossfeldiana* was added to the cells at concentrations of 30, 60, and 100 µg/mL. The control was the cell group with 0.5% DMSO (*v*/*v*). After 24 h, the cells were washed with PBS and stained with the kit reagents from a Muse MitoPotential Assay Kit (Merck Millipore, Burlington, MA, USA), according to the manufacturer’s instruction [53]. A Muse Cell Analyzer (Merck Millipore, Burlington, MA, USA) was used to determine the percentage of live, depolarized live, depolarized dead, and dead cells. All experiments were independently repeated at least two times (in three repetitions).

##### Oxidative Stress Assay

The SKOV-3 and HeLa cells (1 × 10^5^ cells/well) were seeded in 12-well plates and incubated for 24 h. After this time, the water fraction of *K. blossfeldiana* was added to the cells at concentrations of 30, 60, and 100 µg/mL. The control was the cell group with 0.5% DMSO (*v*/*v*). After 24 h, the cells were washed with PBS and stained with the kit reagents from a Muse Oxidative Stress Kit (Merck Millipore, Burlington, MA, USA), according to the manufacturer’s instruction [53]. Analysis was performed with a Muse Cell Analyzer (Merck Millipore, Burlington, MA, USA). The experiments were performed in at least two independent repeats (in three repetitions).

##### Cell Cycle Analysis

The SKOV-3 and HeLa cells (5 × 10^5^ cells/well) were seeded in a 6-well plate and incubated with the water fraction of *K. blossfeldiana* at concentrations of 30, 60, and 100 µg/mL for 48 h. After treatment, the cells were prepared with a Muse Cell Cycle Assay Kit (Merck Millipore, Burlington, MA, USA) and analyzed by a Muse Cell Analyzer (Merck Millipore, Burlington, MA, USA), according to the manufacturer’s instruction [53]. The experiment was repeated in two independent repeats (in three repetitions).

### 4.3. Microbiological Study

#### 4.3.1. Microorganism Species

Gram-positive bacteria: *Streptococcus β*-hemolyzing group A PCM465 (PCM—Polish Collection of Microorganisms), *Streptococcus β*-hemolyzing group G ZMF (collection of the Department of Pharmaceutical Microbiology, Medical University of Gdańsk), *Corynebacterium diphtheriae* ZMF (collection of the Department of Pharmaceutical Microbiology, Medical University of Gdańsk), *Staphylococcus aureus* ATCC6538, *Staphylococcus epidermidis* ATCC14990, *Clostridium sporogenes* ATCC19404, *Clostridium bifermentans* ATCC638, *Cutibacterium acnes* ATCC6919, and *Streptococcus equinus* ATCC15351. Gram-negative bacteria: *Helicobacter pylori* ATCC43504. Yeast: *Candida albicans* ATCC10231. Brain–heart infusion broth (BHI, Becton Dickinson, Franklin Lakes, NJ, USA) was supplemented with 10% bovine serum for *Clostridium sporogenes* ATCC19404, *Clostridium bifermentans* ATCC638, *Cutibacterium acnes* ATCC6919, and *Streptococcus equinus* ATCC15351 (strain growth in GENbag anaerobe, BioMerieux at 37 °C for 48 h). *Staphylococcus epidermidis* ATCC14990, *Staphylococcus aureus* ATCC6538 was grown in Mueller–Hinton broth (MH cation-adjusted, Becton Dickinson, Franklin Lakes, NJ, USA) in an aerobic atmosphere at 37 °C for 48 h. *Corynebacterium diphtheriae* ZMF was grown in brain–heart infusion broth (BHI, Becton Dickinson, Franklin Lakes, NJ, USA) supplemented with 10% bovine serum in an aerobic atmosphere at 37 °C for 72 h. *Streptococcus β*-hemolyzing group A PCM465, *Streptococcus β*-hemolyzing group G ZMF was grown in BHI (Becton Dickinson, Franklin Lakes, NJ, USA) supplemented with 10% bovine serum in GENbag CO_2_, BioMerieux at 37 °C for 48 h. *Helicobacter pylori* was incubated in BHI (Becton Dickinson, Franklin Lakes, NJ, USA) supplemented with 10% horse serum in GENbag microaer, BioMerieux at 37 °C for 72 h. *Candida albicans* was grown in Sabourauda broth (Sb, Becton Dickinson, Franklin Lakes, NJ, USA) in an aerobic atmosphere at 37 °C for 72 h. After determination of the bacterial viability, BHI blood agar plates, MH agar plates, and Sb agar plates were used.

#### 4.3.2. Antibacterial Assay

Active cultures for experiments were prepared by transferring cells from the stock cultures to tube with adequate broth, as described above. They were incubated without agitation for 24 h at 37 °C. The cultures were diluted with adequate broth to achieve an optical density corresponding to 10^6^ colony-forming units per mL (CFU/mL) for bacteria species and 10^3^ CFU/mL for *Candida albicans* (except *Corynebacterium diphtheriae* ZMF, *Cutibacterium acnes*, *Clostridium sporogenes*, *Clostridium bifermentans*, *Streptococcus equinus*, and *Helicobacter pylori*). For *Corynebacterium diphtheriae* ZMF, *Cutibacterium acnes* [54]*, Clostridium sporogenes* [55], *Clostridium bifermentans, Streptococcus equinus*, and *Helicobacter pylori*, inoculum was prepared from bacterial colonies grown on BHI blood agar plates that had been incubated for 48 h in appropriate conditions, with final inoculum concentration of approximately 10^6^ CFU/mL.

The minimum inhibitory concentration (MIC) was determined by broth microdilution technique using 96-well plates [56]. After filling each well with 100 μL of broth, dry test samples of the plant fractions were dissolved in dimethyl sulfoxide (DMSO). These solutions were diluted and added to the first well of each microtiter line. Dilution in geometric progression were carried out by transferring the mixture/dilution (100 μL) from the first to the twelfth well. An aliquot (100 μL) was discarded from the twelfth well. The final concentrations of the extracts and reference ampicillin (for bacteria) and ketoconazole (for yeast) used in the antimicrobial experiments ranged from 9 to 0.0035 mg/mL, from 128 to 0.0625 μg/mL, and from 125 to 0.0625 μg/mL, respectively. Tests were incubated in adequate conditions, as described above. The end point was made by visual observation of growth. The MIC was considered as the lowest sample’s concentration that prevented visible growth. In addition, 100 μL of suspension from each well without growth was inoculated in agar plates to control bacterial viability. After 48 h of incubation, plates were observed for bacterial growth. The MBC (minimal bactericidal concentration) was defined as the minimum concentration of the fractions required to kill the organisms.

### 4.4. Antioxidant Tests

#### 4.4.1. DPPH Assay

The radical scavenging activity of the water fraction of *K. blossfeldiana* was determined by DPPH decolorization method [57]. First, 30 µL of different concentrations of the fraction were dissolved in DMSO and were mixed with 170 µL of 0.06 mM DPPH (methanolic solution). After 40 min of incubation at room temperature in the dark, the absorbance was analyzed at λ = 510 nm by a 96-well microplate reader (Epoch, BioTek System, Winooski, VT, USA). DPPH solution with DMSO was used as a control. The ascorbic acid was used as a standard.

DPPH inhibition was calculated as follows:

DPPH Inhibition (%) = [(Control − Sample)/Control] ×100%(1)

IC_50_ values of the samples were calculated with the program GraFit v. 7.0 (Erithacus Software, East Grinstead, West Sussex, UK). The analysis was performed in triplicate, three replications in each (*n* = 9).

#### 4.4.2. ABTS Assay

The radical scavenging activity of the water fraction of *K. blossfeldiana* was determined by ABTS decolorization method [58]. First, 30µL of different concentrations of the fraction were dissolved in DMSO and mixed with 170 µL of ABTS solution (2 mM ABTS diammonium salt, 3.5 mM potassium persulfate), followed by 10 min incubation at 30 °C in the dark. The absorbance was analyzed at λ = 750 nm by a 96-well microplate reader (Epoch, BioTek System). ABTS solution with DMSO was used as a control. The ascorbic acid was used as a standard.

ABTS inhibition was calculated as follows:

ABTS Inhibition (%) = [(Control − Sample)/Control] × 100%(2)

IC_50_ values of the samples were calculated with the program GraFit v. 7.0 (Erithacus Software, East Grinstead, West Sussex, UK). The analysis was performed in triplicate, three replications in each (*n* = 9).

### 4.5. Phytochemical Study of K. blossfeldiana and K. pinnata Water Fractions

Commercial reference standards used for semi-quantitative method calibration: derivatives of anthraquinones (aloe–emodin, aloe–emodin-8-Glu, emodin, and emodin-8-Glu, rhein), stilbenes (resveratrol, pterostilbene, pinostilbene, astringin, polydatin, rhapontigenin, rhaponticin, isorhapontigenin deoxyrhapontigenin, and deoxyrhaponticin), phenolic acids (glucogallin and gallic acid), flavones (vicenin-II, vicenin-III, and apigenin-7-Glu), flavanones (pinocembrin), chalcones (phloretin), catechins (catechin, epicatechin, gallocatechin, procyanidin-B2, procyanidin-B3, and procyanidin-C1), tryptophan, phenylalanine, formic acid, and LC-MS grade acetonitrile were purchased from Merck (Darmstadt, Germany). Ultrapure water was prepared using a Milli-Q water purification system (MerckMillipore). Malonyl glucosides of kaempferol and quercetin were isolated from the leaves of *Pulmonaria officinalis* [59].

Samples for UHPLC-MS analyses were prepared following a simplified protocol of Salem et al. [60]. Briefly, dried fractions samples (5 mg, weighted with 0.1 mg accuracy) were incubated with 1 mL of methyl–tertbutyl ether/methanol mixture (3:1) for 45 min at 4 °C. Following 15 min of sonication in an ice water bath, 0.7 mL of distilled water/methanol mixture (3:1) was added, and phases were separated by centrifugation (21,000× *g*, 20 min, 4 °C). The water/methanol phase was collected, evaporated under a stream of nitrogen at 30 °C, and reconstituted in 200 µL of 40% methanol. Before the analysis, reconstituted samples were filtered through 0.2 µm cellulose centrifugal filters.

UHPLC-MS analyses were carried out on a Thermo Ultimate 3000 RS chromatographic system coupled/hyphenated with a Bruker Impact II HD (Bruker, Billerica, MA, USA) quadrupole time-of-flight (Q-TOF) mass spectrometer. Analytes were chromatographed on a Waters HSS T3 column (150 mm × 2.1 mm, 1.8 µm, Milford, MA, USA) with the appropriate pre-column. The separations were performed at 45 °C, with a constant flow rate of 0.4 mL/min. The mobile phase A was 0.1% (*v*/*v*) formic acid, and the mobile phase B was acetonitrile with 0.1% (*v*/*v*) of formic acid. The elution profile started isocratically with 5% of phase B for 1 min, followed by linear gradients from 5 to 14% over 7.5 min and 14 to 48% of phase B over 18.5 min. Following that, the concentration of the mobile phase was increased over 4.5 min to 95% of phase B, held at that level for 7 min, and then equilibrated for 5 min with 5% of phase B.

The flow from the column was split between the charged aerosol detector (Thermo Corona Veo RS) and the mass spectrometer type QTOF (Bruker Impact II HD) ion source in a 3-to-1 ratio using a fixed flow splitter. The electrospray ionization ion source operated with the following parameters: voltage capillary 3.0 kV; nebulizer pressure 0.6 bar; drying gas flow 6 L/min; drying gas temperature 200 °C; ion energy 4 eV; RF collision cell 700.0 V(pp); transfer time 100.0 μs; and pre-pulse storage 10.0 μs. Negative ions were measured in the m/z (mass-to-charge ratio) range of 100–1200, with a 5 Hz scanning frequency. MS/MS spectra were obtained in data-dependent mode, in which two of the most intense ions were fragmented by collision-induced dissociation (CID, Ar collision gas). The collision energies for fragmentation were set to 10, 20, and 30 eV in each subsequent analysis of the given sample. The precursor isolation width was kept constant at 4 mass units to obtain sufficient amounts of isotopic data for chemical formula calculations. Internal mass calibration for quadrupole and TOF analyzers was based on lithium formate clusters automatically injected in a 10 mM solution in 50% methanol into the ion source immediately before each analysis.

Absolute quantitation of all analytes was impossible due to the lack of commercially available reference standards. Wherever possible, we semi-quantified observed analytes on the basis of the signal from CAD. The dependence of the signal intensity on the mobile phase composition was established using 20 reference standards differing in retention time and, therefore, the CAD response at the same concentration. Each reference standard was analyzed at 5 concentration levels (from 0.5 to 50 µg/mL) to obtain calibration curves, as proposed previously [61]. The concentration of each analyte was then calculated as a weighted average of the responses of the pair of adjacent calibration standards with RT lower and higher than the RT of the analyte. Weights were calculated on the basis of RT differences between the selected calibration standards and the analyte.

Combined samples spiked with 20 reference standards at two concentration levels (3.5 and 35 µg/mL) were used as quality control samples and analyzed after each block of 10 injections of regular samples. The Bruker Data Analysis, version 4.4 SR1, was used for data analysis and processing. Preliminary identification of metabolites was performed using high-resolution m/z measurements, with errors not exceeding 5 ppm. On the basis of these results, chemical formulas were calculated. The agreement of these formulas with structural insights gained from MS/MS spectra was validated by computational methods using SIRIUS ver. 5.6.2 software [62].

Identification of detected compounds was based mainly on comparisons with reference spectra and MS/MS spectra interpretation aided by SIRIUS and MetFrag [63].

Each tentatively identified metabolite was assigned an identification confidence level class, as suggested by Schrimpe-Rutledge et al. [64]. The Category 1 metabolites were confirmed using authentic reference standards, whereas Category 4 compounds were assigned only elemental sum formulas and had no identifiable structural features in their MS/MS spectra.

### 4.6. Statistical Analysis

Statistical analysis was performed with the STATISTICA 12.0 software package (StatSoft. Inc., Tulsa, OK, USA). All data are expressed as mean values ± standard deviation (SD). The Student’s *t*-test was used to compare the results with the control sample. The statistical significance was set at *p* < 0.05.

## 5. Conclusions

In this work, we estimated the cytotoxic and antimicrobial activity of water and dichloromethane fractions of *K. daigremontiana*, *K. pinnata*, and *K. blossfeldiana.* The water fraction of *K. blossfeldiana* was active both on the cancer cells and *Staphylococcus* bacteria strains. This fraction showed significant cytotoxic effect on the SKOV-3 and HeLa cells, triggered depolarization of the mitochondrial membrane potential, and cell cycle arrest in the G2/M phase in both cell lines. The fraction did not significantly increase the oxidative stress level in the cancer cells and showed strong antioxidant activity in the in vitro tests. The water fraction of *K. blossfeldiana* may be used potentially in the prevention and treatment of cancers and bacterial infections, although the literature does not describe the use of *K. blossfeldiana* in traditional medicine. Further studies should be conducted on the possibility of using this plant as a valuable medicinal raw material. On the other hand, the antimicrobial activities of *K. pinnata* and *K. daigremontiana*, described in this work, corroborate their wide range of applications in various diseases, such as chronic bronchitis, pneumonia, otitis, urethritis, arthritis, and eye and skin infections.

## Figures and Tables

**Table 1:** The IC_50_ (µg/mL) values of the *Kalanchoe* fractions obtained on different cancer cell lines in MTT assay.

	*K. b.* Water Fraction	*K. p.* Water Fraction	*K. d.* Water Fraction *	*K. b. * Dichlorometh. Fraction	*K. p.* Dichlorometh. Fraction	*K. d. * Dichlorometh. Fraction *	Vinblastine Sulphate
HeLa	28.28 ± 2.76	79.91 ± 1.82	>100	>100	>100	6.42 ± 0.34	0.005 ± 0.0004
SKOV-3	32.51 ± 0.69	64.89 ± 1.85	>100	>100	32.10 ± 0.73	5.42 ± 0.16	0.008 ± 0.0003
MCF-7	>100	>100	>100	>50	>100	8.02 ± 0.13	0.007 ± 0.0008
A375	49.44 ± 2.43	>100	>100	95.11 ± 6.35	49.7 ± 1.30	7.72 ± 0.44	0.008 ± 0.0003

*K.b.*—*Kalanchoe blossfeldiana*, *K.p.*—*Kalanchoe pinnata*, *K.d.*—*Kalanchoe daigremontiana*, dichlorometh.—dichloromethane. The mean values ± standard deviation (±SD) were obtained from three independent experiments (in six repetitions, *n* = 18). * These results have been described in the previous work [22].

**Table 2:** Antioxidant activity of the water fraction of *Kalanchoe blossfeldiana*.

IC_50_ (µg/mL)		
	Water Fraction of *K. blossfeldiana*	Ascorbic Acid
DPPH	9.44 ± 0.06	13.1 ± 0.17
ABTS	3.17 ± 0.1	5.26 ± 0.04

The results are presented as mean values ± standard deviation (±SD) from three independent repeats (in three repetitions, *n* = 9).

**Table 3:** The MIC and MBC (mg/mL) values of the *Kalanchoe* sp. fractions on bacteria strains and *Candida albicans*.

Microorganism Species	*K. blossfeldiana* Water Fraction		*K. pinnata* Water Fraction		*K. daigremontiana* Water Fraction		*K. blossfeldiana* Dichlorometh. Fraction		*K. pinnata* Dichlorometh. Fraction		*K. daigremontiana* Dichlorometh. Fraction		Ampicillin	Ket.
	MIC	MBC	MIC	MBC	MIC	MBC	MIC	MBC	MIC	MBC	MIC	MBC	MIC	MIC
*S.* β-hemolyzing group A PCM465	>1.0	>1.0	>2.0	>2.0	4.5	9.0	5.0	5.0	2.0	>2.0	2.0	2.0	3.13 × 10^−4^	n.t.
*S.* β-hemolyzing group G	>1.0	>1.0	>2.0	>2.0	9.0	9.0	>5.0	>5.0	2.0	>2.0	2.0	2.0	1.6 × 10^−4^	n.t.
*C. diphtheriae*	>1.0	>1.0	>2.0	>2.0	4.5	9.0	5.0	5.0	>2.0	>2.0	2.0	2.0	1.6 × 10^−4^	n.t.
*S. aureus* ATCC6538	0.032	>1.0	0.064	>2.0	2.25	>9.0	>5.0	>5.0	2.0	>2.0	>2.0	>2.0	8 × 10^−5^	n.t.
*S. epidermidis* ATCC14990	0.016	>1.0	0.032	>2.0	0.064	>9.0	0.064	>5.0	2.0	>2.0	>2.0	>2.0	3.13 × 10^−4^	n.t.
*H. pylori* ATCC43504	>1.0	>1.0	>2.0	>2.0	>9.0	>9.0	1.25	>5.0	15	>15	>2.0	>2.0	3.2 × 10^−3^	n.t.
*C. acnes* ATCC6919	0.5	>0.5	1.0	>1.0	4.5	>4.5	>2.5	>2.5	15	>15	1.0	>1.0	0.032	n.t.
*S. equinus* ATCC15351	0.5	>0.5	1.0	>1.0	4.5	>4.5	>2.5	>2.5	15	>15	1.0	>1.0	0.016	n.t.
*C. bifermentans* ATCC638	0.25	>0.5	1.0	>1.0	4.5	>4.5	2.5	>2.5	1	>15	0.25	>1.0	0.016	n.t.
*C. sporogenes* ATCC19404	0.5	>0.5	1.0	>1.0	4.5	>4.5	2.5	>2.5	2	>15	0.125	>1.0	<6.3 × 10^−5^	n.t.
*C. albicans* ATCC10231	>1.0	>1.0	>2.0	>2.0	9.0	9.0	5.0	5.0	2.0	>2.0	2.0	2.0	n.t.	>0.125

MIC—minimal inhibitory concentration, MBC—minimal bactericidal concentration, Ket.—ketoconazole, n.t.—not tested.

**Table 4:** Compound categories detected in the water fractions of *Kalanchoe blossfeldiana* and *Kalanchoe pinnata* by LC-QTOF-MS.

Compound Categories	*K. blossfeldiana* Water Fraction	*K. pinnata* Water Fraction	Total Number of Compounds in Each Group
Unidentified	55	26	68
Flavonol glycoside	8	25	31
Acyclic alcohol glycoside	9	14	16
Benzoic acid derivative	8	12	14
Phenylpropanoid derivative	8	11	13
Gallic acid derivative	12	6	13
Organic acid	11	12	12
Megastigmane glycoside	4	10	10
Flavanol	7	0	7
Dimeric proanthocyanidin	6	2	6
Acyclic nitrile glycoside	3	5	5
Phenol derivative	2	1	2
Aminoacid	3	3	3
Acyclic acid glycoside	2	0	2
Phenylethane glycoside	2	0	2
Monoterpene derivative	2	0	2
Iridoid glycoside	1	1	2
Carbohydrate	1	1	1
Acetophenone derivative	1	1	1
Sesquiterpenoid derivative	1	1	1
Dimeric iridoid derivative	1	0	1
Flavanone derivative	0	1	1
Bicyclo[3.1.1] glycoside	1	0	1
Dihydrochalcone derivative	1	0	1
Fatty acid glycoside	1	0	1
Flavonol	0	1	1
Lipid	1	1	1

## Data Availability

Not applicable.

## Supplementary Materials

The following supporting information can be downloaded at: https://www.mdpi.com/article/10.3390/plants12122268/s1; Table S1: The phytochemical analysis of the water fractions of *Kalanchoe blossfeldiana* and *Kalanchoe pinnata* performed by LC-QTOF-MS. Figure S1: Phytochemical profiles of the water fraction of *Kalanchoe blossfeldiana* obtained by LC-QTOF-MS. Figure S2: Phytochemical profiles of the water fraction of *Kalanchoe pinnata* obtained by LC-QTOF-MS.
