# Supplementary material for: Kalanchoe sp. Extracts—Phytochemistry, Cytotoxic, and Antimicrobial Activities
Source: Plants (Basel). 2023 Jun 10;12(12):2268. doi: 10.3390/plants12122268 (PMC10305294; doi:10.3390/plants12122268)
Supplement: Supplementary file 1 [file plants-12-02268-s001.zip › Figure S1 and S2 Supplementary materials.pdf]

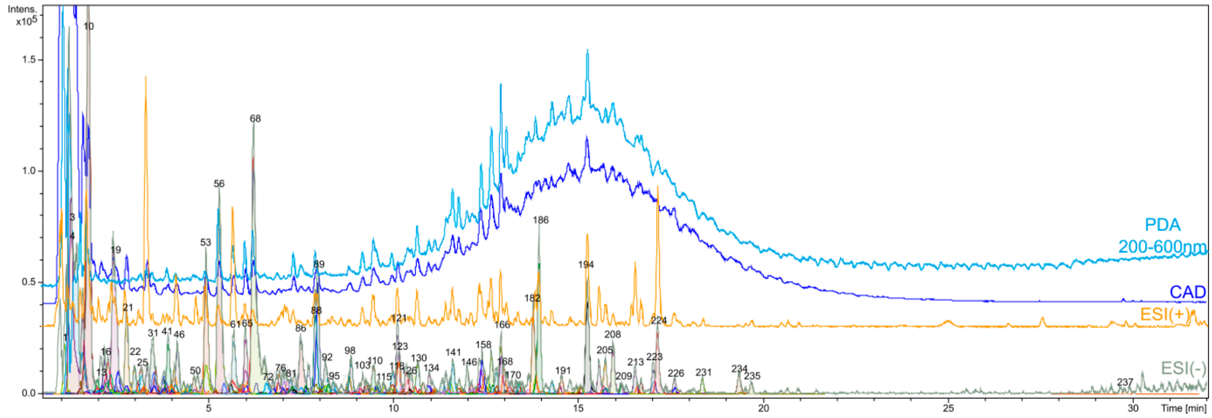

Figure S1. Phytochemical profiles of the water fraction of *Kalanchoe blossfeldiana* obtained by LC-QTOF-MS.

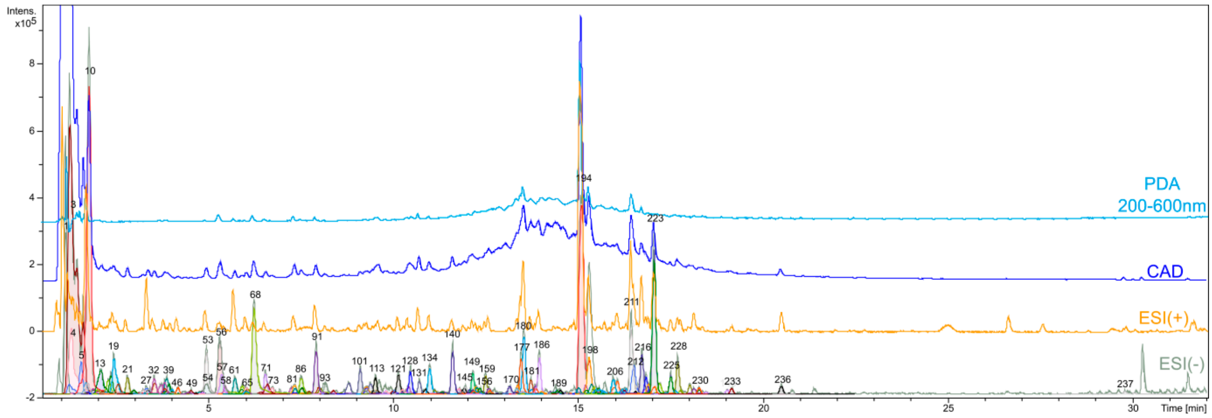

Figure S2. Phytochemical profiles of the water fraction of *Kalanchoe pinnata* obtained by LC-QTOF-MS.
